# 170. Outcomes after *Stenotrophomonas maltophilia* bloodstream infection

**DOI:** 10.1093/ofid/ofad500.243

**Published:** 2023-11-27

**Authors:** Maha Y Al-Jabri, Maria F Mojica, Dylan Brown, Kaleb H Wolfe, Giusy Tiseo, Valentina Galfo, Gauri Rao, Robert A Bonomo, Marco Falcone, David van Duin

**Affiliations:** Case Western Reserve University - University Hospitals Cleveland Medical Center, Cleveland, Ohio; Case Western Reserve University, Cleveland, Ohio; University of North Carolina at Chapel Hill, Chapel Hill, North Carolina; Vanderbilt University Medical Center, White House, Tennessee; Infectious Diseases Unit/University of Pisa, Pisa, Toscana, Italy; University of Pisa, Pisa, Toscana, Italy; UNC Eshelman School of Pharmacy, NC; Case Western Reserve University, Cleveland, Ohio; Infectious Diseases Unit/University of Pisa, Pisa, Toscana, Italy; University of North Carolina at Chapel Hill, Chapel Hill, North Carolina

## Abstract

**Background:**

*Stenotrophomonas maltophilia* is a leading cause of carbapenem-resistant, Gram-negative bacterial bloodstream infections (BSI).

**Methods:**

A retrospective, observational study was conducted at four centers in Italy and the United States. Patients who were treated for *S. maltophilia* BSI between 1/1/2015 through 12/31/2020 with available antimicrobial susceptibility testing (AST) as reported by the local clinical microbiology laboratories were included. Data extracted from the electronic medical record and collected in a central database. Acute critical illness was measured through the Pitt Bacteremia Score, and chronic comorbidities through the Charlson Comorbidity Index. Desirability of outcome ranking (DOOR) outcomes were determined as shown.

Table.

**Figure:**
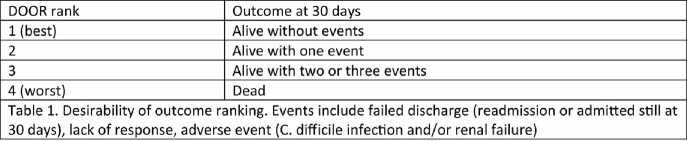

DOOR Outcomes

**Results:**

Median age of 143 patients was 54 years (IQR 40-65 years), and 77/143 (54%) were women. The median Charlson Comorbidity Index and Pitt bacteremia score were 3 (IQR 2-5), and 1 (IQR 0-2). At time of culture, 22/143 (15%) patients were in the intensive care unit, and 15/143 (10%) patients were on mechanical ventilation. Of tested isolates, 4/142 (3%), 0/53 (0%), 17/140 (12%) were non-susceptible to trimethoprim/sulfamethoxazole, minocycline, and levofloxacin, respectively. In the first 14 days after culture, 68/143 (48%) received at least 2 different antibiotics either together or sequentially. Levofloxacin (84/143, 59%), trimethoprim/sulfamethoxazole (68/143, 48%), and tetracyclines (23/143, 16%) were most used. Novel treatment approaches were less commonly employed; cefiderocol in 5/143 (3%), and ceftazidime-avibactam & aztreonam in 6/143 (4%) of patients. Overall mortality at 30 days was 29/143 (20%); 30-day mortality was 8/17 (47%) in patients with fluoroquinolone-non-susceptible isolates, vs. 20/123 (16%) in patients with fluoroquinolone-susceptible isolates (p< 0.01). DOOR distribution of outcomes is shown in Figure 1. A randomly selected patient with a fluoroquinolone -susceptible isolate had a 74% (95% CI 61%-84%, p< 0.001) likelihood of a better outcome as compared to a randomly selected patient with a fluoroquinolone-non-susceptible isolate.

**Figure.**
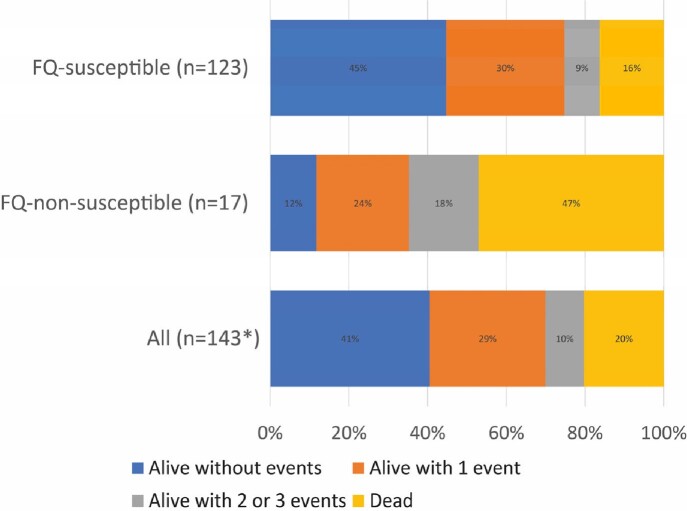
DOOR Outcomes *Three isolates were not tested for fluoroquinolone (FQ) susceptibility

**Conclusion:**

*S. maltophilia* BSI is associated with poor outcomes and high mortality, especially in patients with fluoroquinolone-non-susceptible isolates.

**Disclosures:**

**Giusy Tiseo, MD**, Shionogi: Honoraria **Robert A. Bonomo, MD**, Entasis: Grant/Research Support|Merck: Grant/Research Support|venatorax: Grant/Research Support|Wockhardt: Grant/Research Support **Marco Falcone, MD, PhD**, Gilead: Board Member|Gilead: Honoraria|Menarini: Board Member|Menarini: Grant/Research Support|Menarini: Honoraria|MSD: Board Member|MSD: Grant/Research Support|MSD: Honoraria|Nordic Pharma: Honoraria|Pfizer: Board Member|Pfizer: Honoraria|Shionogi: Honoraria **David van Duin, MD, PhD**, Entasis: Advisor/Consultant|Merck: Advisor/Consultant|Merck: Grant/Research Support|Pfizer: Advisor/Consultant|Pfizer: Honoraria|Qpex: Advisor/Consultant|Roche: Advisor/Consultant|Shionogi: Advisor/Consultant|Shionogi: Grant/Research Support|Union: Advisor/Consultant|Utility: Advisor/Consultant

